# AI-Driven Prediction of Renal Stone Recurrence Following ECIRS: A Machine Learning Approach to Postoperative Risk Stratification Incorporating 24-Hour Urine Data

**DOI:** 10.3390/jcm14124037

**Published:** 2025-06-07

**Authors:** Takahiro Yanase, Rei Unno, Theodoros Tokas, Vineet Gauhar, Yuya Sasaki, Kengo Kawase, Ryosuke Chaya, Shuzo Hamamoto, Mihoko Maruyama, Takahiro Yasui, Kazumi Taguchi

**Affiliations:** 1 Department of Nephro-urology, Nagoya City University Graduate School of Medical Sciences, Nagoya 467-8601, Japan; t_yanase@med.nagoya-cu.ac.jp (T.Y.); unno@med.nagoya-cu.ac.jp (R.U.); kawase@med.nagoya-cu.ac.jp (K.K.); brutus.you.are.fool.r.c@gmail.com (R.C.); hamamo10@med.nagoya-cu.ac.jp (S.H.); yasui@med.nagoya-cu.ac.jp (T.Y.); 2 Department of Urology, University General Hospital of Heraklion, Medical School, University of Crete, 71500 Heraklion, Greece; ttokas@yahoo.com; 3 Training and Research in Urological Surgery and Technology (T.R.U.S.T.)-Group, 6060 Hall in Tirol, Austria; 4 Ng Teng Fong General Hospital, (NUHS), Singapore 609606, Singapore; vineetgaauhaar@gmail.com; 5 Asian Institute of nephrourology (AINU), Hyderabad 500082, India; 6 Graduate School of Information Science and Technology, University of Osaka, Osaka 565-0871, Japan; sasaki@ist.osaka-u.ac.jp; 7 Graduate School of Engineering, University of Osaka, Suita, Osaka 565-0871, Japan; maruyama@eei.eng.osaka-u.ac.jp; 8 Department of Urology, University of Alabama at Birmingham, Birmingham, AL 35294, USA

**Keywords:** kidney stone recurrence, artificial intelligence, machine learning, 24 h urine collection, endoscopic combined intrarenal surgery (ECIRS)

## Abstract

**Background/Objectives**: Predicting kidney stone recurrence after active stone treatment remains challenging due to its multifactorial nature. Artificial intelligence, particularly machine learning, provides new methods for identifying hidden patterns in high-dimensional clinical data. We conducted a study applying machine learning to identify key predictors of recurrence following endoscopic combined intrarenal surgery (ECIRS) **in patients with calcium stones. Methods**: This retrospective cohort analysis included 72 patients with calcium stones who underwent ECIRS between June 2019 and May 2022 and achieved a complete stone-free status on postoperative CT. Patients were followed for two years, with recurrence assessed through protocolized imaging. We collected 235 variables, including clinical data, 24 h urine collections, stone composition, imaging features, and perioperative findings. Several machine learning models were developed, and SHapley Additive exPlanations (SHAP) analysis identified features associated with recurrence. **Results**: Within two years, 29 of 72 patients (40.3%) experienced recurrence. The TabNet model demonstrated the highest predictive accuracy (AUC = 0.89), outperforming traditional machine learning algorithms. SHAP analysis identified urinary oxalate ≥ 25.4 mg/day and hemoglobin (Hb) drop ≥ 0.3 g/dL at 3 months postoperatively as independent predictors, even within normal limits. A simplified TabNet-based model using three key features (oxalate, urine volume, and 3-month ΔHb) maintained a strong performance (AUC = 0.75), supporting its clinical utility. **Conclusions**: Machine learning enabled the accurate prediction of kidney stone recurrence after ECIRS. The inclusion of 24 h urine data significantly improved the performance. Even patients with “normal” oxalate levels showed increased risk, suggesting current clinical thresholds may require re-evaluation.

## 1. Introduction

Endourologists have increasingly recognized the global rise in kidney stone disease, fueled by expanding clinical databases, electronic medical records, and worldwide surveillance efforts [1]. The prevalence varies in regions, but a recent cross-sectional cohort revealed that 11% of men and 7% of women had reported a lifetime history [2]. In addition, the annual incidence of kidney stone disease approached 1% in 2015, which increased from 0.6% in 2005 [3]. This trend contributed to the increased number of surgical treatments we encountered in our daily practice, including ureteroscopy (URS) and percutaneous nephrolithotomy (PCNL) [4,5]. However, the number of cases again requires additional surgical treatment owing to the clinically significant residual fragments and recurrence of stones from months to years after the initial treatments.

Stone recurrence rates remain high—approximately 50% within 5 years and up to 80–90% within 10 years [6,7]. Risk accumulates with each recurrence and surgical patients, who often present with larger or more complex stones, are particularly vulnerable. A wide range of factors, including a younger age, male sex, high BMI, family history, prior stone episodes, hypertension, and prior surgery, have been associated with recurrence [8,9]. Yet despite these insights, only 3% of patients receive metabolic evaluations (including 24 h urine collections) or medications after a single episode [10], underscoring the need for improved risk stratification. An accurate recurrence prediction allows early, tailored interventions for high-risk individuals while sparing low-risk patients from unnecessary testing or treatment.

Patients undergoing PCNL often have large, complex renal stones and are characterized by risk factors such as prolonged metabolic abnormalities; if these underlying causes remain unaddressed, the postoperative recurrence risk remains high [11]. While several studies have assessed the prediction of surgical outcomes such as their stone-free status and complication rate by nomograms and software analysis, recurrence prediction is challenging due to numerous factors that could individually affect perioperative outcomes. Considering the recent remarkable development of artificial intelligence (AI) applications in the diagnostic and treatment field in endourology [12], the AI usage might be a solution for prediction models for the recurrence of complex kidney stones.

Traditional statistical models, such as logistic regression, have been used to estimate recurrence risk, but are limited by their assumption of linear relationships between predictors and outcomes. In contrast, machine learning (ML) algorithms can capture complex, non-linear interactions among variables, allowing for the identification of patterns that may be overlooked by conventional methods. These non-linear associations are particularly relevant in multifactorial conditions such as stone recurrence, where subtle interactions among metabolic, anatomical, and clinical factors may contribute synergistically to risk. By leveraging ML, it is possible to improve the predictive accuracy and uncover previously unrecognized relationships that inform clinical decision making. In this study, we conducted a retrospective cohort analysis for the postoperative recurrence of renal stones after endoscopic combined intrarenal surgery (ECIRS) for large renal stones to develop an AI prediction model.

## 2. Materials and Methods

### 2.1. Study Population and Inclusion Criteria

**Figure 1:**
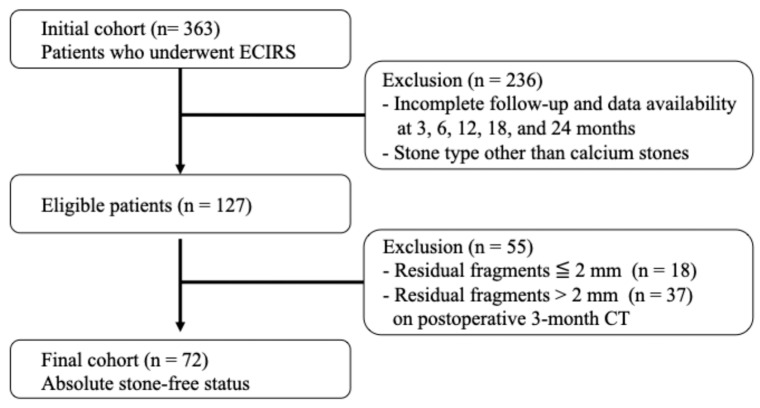
Flowchart of patient selection.

This retrospective analysis utilized prospectively collected clinical data and enrolled patients who underwent ECIRS at Nagoya City University Hospital between June 2019 and May 2022. All patients for whom ECIRS was considered the primary surgical approach, per the European Association of Urology and American Urological Association guidelines, were eligible for this study. ECIRS is often chosen for managing large, complex, or staghorn calculi due to its combined antegrade and retrograde access, which improves stone clearance and reduces the need for secondary procedures. In addition to clinical suitability, the final decision to proceed with ECIRS was made based on patient preference following a detailed explanation of available options. Among the 363 patients initially treated with ECIRS, a total of 127 patients were ultimately included in the analysis based on strict protocol-defined criteria. The patient selection process is illustrated in Figure 1. The inclusion criteria were as follows: (1) complete stone-free status confirmed by non-contrast-enhanced CT with 1.5 mm slices at 3 months postoperatively; (2) stone composition limited to calcium oxalate (CaOx) or calcium phosphate (CaP); and (3) full data availability at all protocol-defined follow-up points (3, 6, 12, 18, and 24 months). Protocol-based clinical visits, laboratory tests, CT scans, and 24 h urine collections were completed without missing data. Exclusion criteria included patients < 18 years, pregnancy, inability to provide informed consent, history of ileal conduit or other urinary tract modification, severe urinary tract infection, coagulopathy, American Society of Anesthesiologists physical status classification ≥III, or severe ureteral strictures.

From this cohort, 363 patients underwent ECIRS for renal stones at Nagoya City University Hospital between January 2019 and May 2022. After applying the inclusion criteria, (1) complete stone-free status confirmed by 1.5 mm slice non-contrast-enhanced CT at 3 months postoperatively, (2) stone composition limited to calcium oxalate (CaOx) or calcium phosphate (CaP), and (3) full availability of clinical, imaging, and laboratory data at all predefined follow-up points—a total of 72 patients were included in the final analysis.

### 2.2. Definition of Recurrence and Imaging

Stone recurrence was defined as the presence of newly formed calculi detected by non-contrast-enhanced computed tomography (CT) at either 1 year or 2 years following surgery. To ensure accurate identification of postoperative recurrence, all patients included in the analysis had previously achieved complete stone clearance, confirmed by non-contrast CT performed 3 months postoperatively. Patients who demonstrated any residual stone fragments at the 3-month postoperative CT, regardless of fragment size, were excluded from this recurrence analysis. All CT scans were conducted using a standardized low-dose imaging protocol with 1.5 mm slice thickness to minimize radiation exposure while maintaining sufficient image resolution. In patients with severe obesity or suboptimal image quality, a standard-dose CT protocol was employed to enhance diagnostic accuracy. Image evaluation for recurrence was independently performed by two board-certified urologists. Discrepancies in interpretation were resolved by consensus, ensuring a robust and reproducible assessment of stone recurrence.

### 2.3. Surgical Procedure

All surgeries were performed using a standardized ECIRS protocol. Patients were placed in either the Galdakao-modified supine Valdivia or prone split-leg position to enable simultaneous retrograde intrarenal surgery and percutaneous nephrolithotomy (PCNL) by two urologists. Retrograde procedures involved laser lithotripsy with a 200 or 272 μm Holmium:YAG laser (Cyber Ho^®^, Quanta System, Milan, Italy), with either dusting or fragmentation mode selected at the surgeon’s discretion. Swiss LithoClast^®^ Master (Electro Medical Systems, Nyon, Switzerland) was used for stone fragmentation on the PCNL side. A ureteral access sheath (10/12 F to 12/14 F) was employed when necessary. All patients received a 6 F double-J ureteral stent postoperatively, which was routinely removed one month after surgery. Detailed surgical techniques are described in a previous publication [13].

### 2.4. Data Collection and Variables

A total of 235 features were collected per patient. These included 74 patient background variables: age, sex, body mass index (BMI), performance status, comorbidities, and medication use; 10 stone questionnaire items: family history, previous treatments, and age at onset; 75 laboratory parameters: including preoperative, postoperative day 1, and 3-month blood and spot urine results; 12 24-h urine parameters: including volume, calcium, oxalate, phosphate, uric acid, sodium, creatinine, and supersaturation levels for CaOx and CaP, performed at 6 months postoperatively; and 20 CT imaging features derived from preoperative CT scans: including stone size, number of involved calyces, stone density in Hounsfield Units (HU), and hydronephrosis grade. Additionally, 8 intraoperative variables were recorded, such as surgery time, presence of ureteral damage, and laser use. Stone composition was determined by infrared spectroscopy, and patients were categorized based on the predominant component. In cases of mixed stones, the component with the highest relative proportion was defined as the primary stone type. Supersaturation levels for CaOx and CaP were estimated using a simplified approximation formula in Python 3, based on urinary calcium, oxalate, phosphate, volume, and pH. EQUIL2 was considered but not used due to missing input data required for accurate computation. All data were de-identified and managed under institutional privacy policies. A complete list of input features is provided in Supplementary Table S1.

### 2.5. Ethics

The study protocol was approved by the Research Ethics Committee of Nagoya City University Hospital (No. 60-19-0044). All participants received comprehensive oral and written explanations regarding this study’s purpose, procedures, risks, and benefits. Written informed consent was obtained from each participant before any study-related activities were initiated. The confidentiality of personal information was strictly maintained and all data were anonymized before analysis to protect participant privacy.

### 2.6. Data Preprocessing

All raw data were subjected to preprocessing to improve model performance and ensure numerical stability. Specifically, three types of preprocessing techniques were considered: (1) no transformation (raw values retained), (2) min–max normalization to scale values in a 0–1 range, and (3) log transformation followed by min–max normalization. These preprocessing methods were applied selectively, depending on the distribution and scale of each variable. Normally distributed variables were either retained as is or normalized. Highly categorical or binary variables were kept in their original form without transformation. No missing data were present, as the study protocol required complete data collection at all follow-up timepoints. All personally identifiable information was removed or anonymized following institutional privacy policies before analysis. This variable-specific preprocessing ensured that each feature was transformed in a manner suitable for its distribution, thereby optimizing model input without distorting inherent relationships in the data.

### 2.7. Baseline Group Comparisons

Baseline characteristics were compared between the recurrence and non-recurrence groups to identify potential differences in demographic, clinical, stone-related, and metabolic parameters. Categorical variables, such as sex and history of previous procedures, were analyzed using the Chi-square test to assess associations between group membership and observed frequencies. For continuous variables, such as age, BMI, and 24 h urinary excretion values, the choice of statistical test depended on the data distribution. Variables that followed a normal distribution were analyzed using the independent samples t-test, while those that did not meet normality assumptions were compared using the non-parametric Mann–Whitney U test. All statistical analyses were two-tailed, and a *p*-value of less than 0.05 was considered to indicate statistical significance.

### 2.8. Prediction Model Development

Multiple supervised ML models were trained using the collected dataset to predict postoperative stone recurrence within 2 years. These included three tree-based models, Random Forest, eXtreme Gradient Boosting (XGBoost), and Light Gradient-Boosting Machine. (LightGBM), and two deep learning models such as Automatic Feature Interaction Learning via Self-Attentive Neural Networks (AutoInt) and Tabular Data Learning Network (TabNet). Additionally, logistic regression was performed to serve as a traditional statistical comparison.

Logistic regression began with univariable analyses to identify significant and clinically relevant variables. Subsequently, multivariable logistic regression was performed with age and sex included as covariates. All models were implemented in Python (version 3.10), and hyperparameter optimization was performed using Optuna. Model training details: After Optuna-driven hyperparameter search, all input features were standardized to mean 0 and variance 1. Class imbalance was addressed by random undersampling of the majority class. Models were trained within a stratified 5-fold cross-validation framework (shuffle = True, random_state = 42).

Tree-based Machine Learning Models: Random Forest (scikit-learn v1.2): An ensemble of bootstrap-aggregated decision trees. Key hyperparameters (n_estimators, max_depth, min_samples_split, and max_features) were tuned via Optuna. XGBoost (xgboost v1.7) and LightGBM (lightgbm v3.3): gradient-boosted tree frameworks. Optuna searched over n_estimators, learning_rate, tree complexity (max_depth for XGBoost; num_leaves for LightGBM), subsampling (subsample/bagging_fraction), feature fraction (colsample_bytree/feature_fraction), and regularization parameters. Early stopping (patience = 20 rounds on validation AUC) was applied in each fold.

Deep Learning Models (AutoInt, TabNet): Both were trained for up to 200 epochs with an initial learning rate of 4 × 10⁻⁴ and batch size of 256, employing early stopping (patience = 20 epochs on validation AUC); the best checkpoint per fold was retained. A utoInt consisted of an input layer, followed by four multi-head self-attention layers, and a fully connected output layer. TabNet employed a sequential attention mechanism across three decision steps.

Model interpretability was enhanced using SHapley Additive exPlanations (SHAP); SHAP values were computed and visualized after final training. Net Reclassification Improvement (NRI) and Integrated Discrimination Improvement (IDI) quantified the incremental value of 24 h urine data, and optimal cutoffs for top SHAP features were determined via Youden’s Index on ROC curves.

### 2.9. Model Evaluation and Comparison

To maintain class balance in each fold, model performance was evaluated using stratified 5-fold cross-validation. Evaluation metrics included area under the receiver operating characteristic curve (AUC), F1 score, sensitivity, and accuracy. DeLong’s test was applied to statistically compare predictive performance between models. Receiver operating characteristic (ROC) curves were also generated to evaluate model discrimination visually. Five predictive models were constructed using different combinations of input features to examine the additive value of specific data types, particularly 24 h urine data. The five combinations models were as follows: (1) blood and urine test (including 24 h urine) with CT, (2) blood and urine test (including 24 h urine), (3) blood and urine test only, (4) a simplified model using a small set of top-ranked features identified by SHAP analysis, and (5) 24 h urine parameters only. Models 1 to 3 included clinical information such as age, sex, and medical history, whereas models 4 and 5 were constructed using a limited number of selected features. Net Reclassification Improvement (NRI) and Integrated Discrimination Improvement (IDI) were calculated to assess risk reclassification due to 24 h urine data. In addition, for the top-ranked features identified through SHAP analysis, optimal cutoff values were determined using Youden’s Index on ROC curves to facilitate clinical interpretation and potential stratification of recurrence risk.

## 3. Results

### 3.1. Patient Characteristics and Recurrence Incidence

Of the 127 patients who met all protocol-based inclusion criteria, 72 (56.7%) achieved an absolute stone-free status at 3 months postoperatively. An additional 18 patients (14.2%) had residual fragments measuring 0–2 mm (relative stone-free), while 37 patients (29.1%) had residual fragments larger than 2 mm. Among the 72 absolute stone-free patients, 29 (40.3%) experienced stone recurrence within two years. Although outside the study cohort, 10 out of 18 patients (55.6%) with 0–2 mm residual fragments showed stone growth to ≥4 mm during the same follow-up period.

### 3.2. Comparison Between Recurrence and Non-Recurrence Groups

Table 1 summarizes the clinical characteristics of the recurrence (*n* = 29) and non-recurrence (*n* = 43) groups among absolute stone-free patients. No significant differences were observed in the sex, age, BMI, stone side, urine volume, or 24 h urine parameters including uric acid, calcium, magnesium, and phosphate (*p* > 0.05).

In contrast, significant differences were observed in age at the first stone episode, history of TUL and PCNL, total stone number, bilateral stone presence, stone composition, and urinary oxalate excretion. Specifically, urinary oxalate excretion was significantly higher in the recurrence group (35.9 ± 19.7 vs. 26.9 ± 17.0 mg/day, *p* < 0.05). Among stone compositions, CaOx was present in 62 patients (20 with recurrence, 32.3%), while CaP was present in 10 patients (9 with recurrence, 90.0%).

### 3.3. Logistic Regression Analysis

As shown in Table 2, univariable logistic regression showed that bilateral stones (OR: 7.02, 95% CI: 1.7–28.5, *p* = 0.006) and a higher stone burden (per 10 mm increase; OR: 1.22, 95% CI: 1.00–1.63, *p* = 0.028) were significantly associated with recurrence.

In multivariable logistic regression, including urinary oxalate excretion as a clinically important variable and adjusting for age and sex, all three variables remained statistically significant: bilateral stones (OR: 12.0, 95% CI: 3.5–41.2, *p* = 0.001), the stone burden (per 10 mm increase; OR: 1.22, 95% CI: 1.10–1.79, *p* = 0.013), and urinary oxalate excretion (per 10 mg/day increase; OR: 1.48, 95% CI: 1.10–2.16, *p* = 0.021).

### 3.4. Model Performance and SHAP Analysis

**Figure 2:**
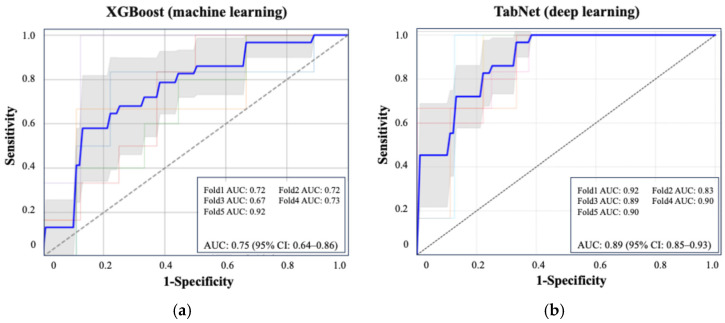
ROC curves of the best-performing machine learning and deep learning models for predicting postoperative stone recurrence. (**a**) XGBoost achieved an average AUC of 0.75 (95% CI: 0.64–0.86). (**b**) TabNet showed superior performance with an AUC of 0.89 (95% CI: 0.85–0.93), based on five-fold cross-validation. Each colored line represents the ROC curve from one fold in five-fold cross-validation. The bold blue line indicates the average ROC curve across all folds.

**Figure 3:**
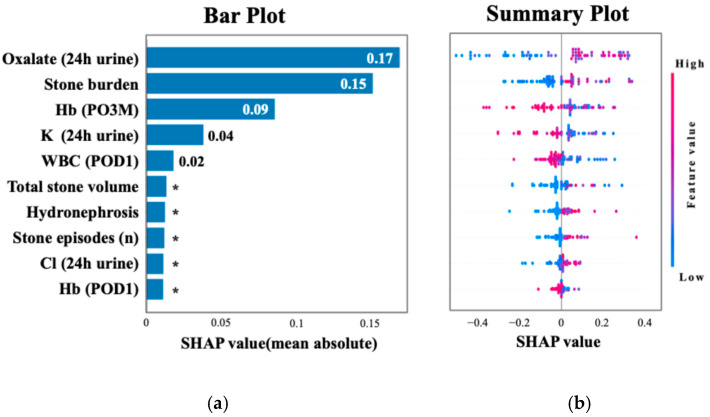
SHAP analysis highlighting key predictors of recurrence. (**a**) The top 10 features ranked by mean absolute SHAP values, with oxalate excretion and stone burden as the most important. (**b**) The distribution of SHAP values colored by feature intensity across patients. Each dot represents a patient, with color indicating the normalized feature value (red = high, blue = low). Higher oxalate excretion (24 h urine), larger stone burden, and lower hemoglobin at 3 months postoperatively were among the strongest predictors of recurrence. * Indicates values less than 0.01.

Figure 2 shows the ROC curve for the XGBoost model, which achieved an AUC of 0.75 (95% CI: 0.64–0.86), and the ROC curve for TabNet showed the highest performance with an AUC of 0.89 (95% CI: 0.85–0.93). Table 3 summarizes the performance metrics of each machine learning algorithm, including the AUC, accuracy, sensitivity, specificity, and F1 score, highlighting the superior discriminative ability of TabNet. Figure 3 displays the top 10 most influential features identified by SHAP value analysis, which was performed using the XGBoost model due to its balance of high interpretability and a competitive predictive performance. The most important predictors were urinary oxalate excretion, the stone burden, and postoperative 3-month hemoglobin (Hb). Notably, only the hemoglobin level measured at 3 months postoperatively showed a strong association with recurrence risk, whereas Hb values on postoperative day 1 and at 1 month were not significantly associated.

**Figure 4:**
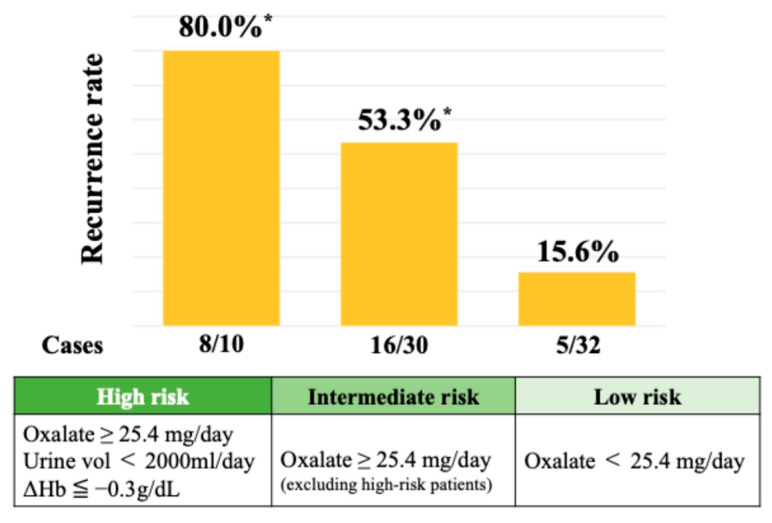
Recurrence rates stratified by urinary oxalate levels and hemoglobin changes. * Chi-square test (*p* = 0.0002).

A SHAP summary plot illustrates how each feature contributes to recurrence risk depending on its value. Higher values of oxalate and stone burden and lower hemoglobin levels were associated with increased risk. Cutoff values for the top three SHAP-ranked features were determined using Youden’s Index. These thresholds were urinary oxalate excretion ≥ 25.4 mg/day, stone burden ≥ 51.0 mm, and postoperative 3-month hemoglobin ≤ 12.9 g/dL. The perioperative hemoglobin change (ΔHb) was also analyzed, given the known sex-related differences in hemoglobin levels. The optimal cutoff for ΔHb was ≤−0.3 g/dL, which was associated with a significantly increased risk of recurrence. As shown in Figure 4, the 2-year recurrence rate reached 80.0% in patients classified as high risk—those with urinary oxalate excretion ≥ 25.4 mg/day, a urine volume < 2000 mL/day, and a hemoglobin decrease (ΔHb ≤ −0.3 g/dL). Among patients with urinary oxalate excretion ≥ 25.4 mg/day who did not meet both the criteria of a low urine volume and hemoglobin decrease (i.e., the intermediate risk group), the recurrence rate remained at 53.3%. In contrast, patients with oxalate excretion < 25.4 mg/day (low-risk group) showed a markedly lower recurrence rate of 15.6%.

Patients with all three risk factors (high oxalate, a low urine volume, and a hemoglobin drop at 3 months postoperatively) showed an 80.0% recurrence rate. Intermediate- and low-risk groups had recurrence rates of 53.3% and 15.6%, respectively.

### 3.5. Comparison of Prediction Models Based on Test Combinations

Five models were constructed using different combinations of clinical and laboratory features to evaluate the contribution of test types.

Model 1: blood + urine test (24 h urine) + CT, AUC = 0.89 (95% CI: 0.85–0.93).
Model 2: blood + urine test (24 h urine), AUC = 0.84 (95% CI: 0.74–0.94).
Model 3: blood + urine test (excluding 24 h urine), AUC = 0.79 (95% CI: 0.57–0.99).
Model 4: oxalate, urine volume, and change in Hb, AUC = 0.75 (95% CI: 0.63–0.87).
Model 5: 24 h urine parameters only, AUC = 0.69 (95% CI: 0.60–0.78).

All models were constructed using the TabNet architecture. Model 1 (blood, 24 h urine, and CT) achieved the highest AUC (0.89), while Model 5 (24 h urine only) had the lowest (0.69). A simplified model using three key features (oxalate, urine volume, and 3-month ΔHb) maintained a strong performance (AUC = 0.75). The ROC curves illustrate the performance across the combinations.

**Figure 5:**
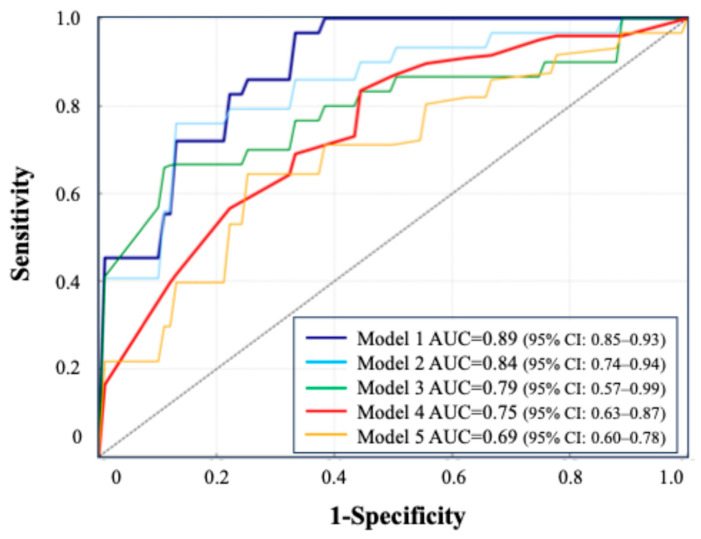
ROC curve comparison based on combinations of clinical tests.

The ROC curves for these five models are presented in Figure 5, illustrating the comparative predictive performance of each data combination. The detailed performance metrics, including the AUC, sensitivity, specificity, and F1 score, are provided in Table 4. Clinical information such as age, sex, and medical history was included in Models 1 to 3.

The simplified model (model 4) achieved a practical predictive performance, despite using only a limited number of variables. In DeLong’s test, Model 1 vs. Model 3 yielded *p* = 0.18, and Model 2 vs. Model 3 yielded *p* = 0.81. Compared to the model excluding 24 h urine data (Model 3), the inclusion of 24 h urine parameters (Model 2) resulted in an NRI of +0.13, indicating that 15% more recurrence cases were correctly reclassified into high-risk categories, with a 2% increase in misclassified non-recurrence cases. The IDI was +0.01. Model 4 showed a superior performance over Model 5, with an NRI of +0.21 (24% improvement in recurrence classification) and an IDI of +0.03.

## 4. Discussion

This study demonstrated that combining rigorous postoperative follow-up, comprehensive metabolic profiling, and advanced machine learning enables the accurate prediction of kidney stone recurrence after ECIRS. Our postoperative model, developed using a strictly defined cohort of patients with a confirmed complete stone-free status, offers notable clinical advantages over existing preoperative tools. Traditional risk models—such as the ROKS nomogram—help estimate the likelihood of recurrence at 5 or 10 years [14]; however, they provide only a general assessment and offer insufficient practical guidance for postoperative care. In contrast, our model identified high-risk individuals immediately after surgical clearance, facilitating prompt preventive intervention and bridging the gap between stone removal and long-term prevention.

In recent years, artificial intelligence (AI), particularly machine learning, has emerged as a transformative tool in urolithiasis. ML-based approaches have demonstrated promising clinical utility. For instance, one study used 24 h urine parameters to predict stone recurrence and achieved a validation AUC of 0.64, highlighting the clinical potential of urine chemistries for risk stratification [15]. Another model detected urinary abnormalities, such as hypercalciuria, without urine collection by using routine clinical data, reporting AUCs of up to 0.79 [16]. A separate ML model classified the stone composition based on electronic health records with an AUC of 0.80, supporting its value in guiding initial treatment decisions [17]. These innovations enable a more precise diagnosis, individualized treatment planning, and risk-adapted follow-up strategies. Our model achieved an AUC of 0.89 using 235 variables, including imaging and 24 h urine data, and outperformed prior efforts using either clinical or urine-based features alone (typically AUCs of 0.63–0.65) [14]. Notably, the model performance declined when limited to 24 h urine data (AUC = 0.69), reinforcing the value of data integration.

Guidelines from the American Urological Association and European Association of Urology endorse 24 h urine testing in high-risk stone formers, citing the urine concentration and volume as modifiable factors in stone formation [18,19]. Nevertheless, interventions based on urine parameters remain underutilized, partly due to uncertainty about actionable values [20]. Our findings suggest that even values within traditional “normal” ranges can carry prognostic significance. Patients with oxalate excretion ≥ 25.4 mg/day were at an elevated risk, despite this threshold falling below the typical hyperoxaluria definition of 40 mg/day [21]. Similarly, mild postoperative anemia, defined as hemoglobin ≤ 12.9 g/dL or a drop ≥ 0.3 g/dL, was associated with recurrence. These findings suggested that conventional cutoffs may miss “subclinical” risks and that personalized thresholds may be warranted, particularly in the postoperative setting. Early counseling or treatment (e.g., dietary oxalate restriction) could be considered for patients with borderline lab values and a history of stones.

The association between anemia and stone recurrence represents a novel pathophysiological insight. Notably, only the hemoglobin level measured at 3 months postoperatively showed a strong association with recurrence risk, whereas levels on postoperative day 1 and at 1 month did not. This temporal pattern suggests that factors beyond perioperative blood loss, such as persistent metabolic derangement or subclinical inflammation, may contribute to the sustained hemoglobin reduction and recurrence risk. The low hemoglobin level might reflect chronic hypoxia in renal tissues, particularly the medulla and papilla, which are already oxygen-deprived under physiological conditions [22]. Reactive oxygen species, which are generated under hypoxic and inflammatory conditions, have been shown to damage renal epithelial cells, promote calcium oxalate crystallization, and facilitate stone retention within the kidney [23]. Alternatively, anemia may reflect underlying chronic inflammation or metabolic disease, conditions also linked to stone risk. Disruptions in iron metabolism may also indirectly influence urinary mineral handling or bone resorption [24,25]. Recent evidence suggests that therapies targeting hypoxia-inducible factors may influence the renal mineral balance and stone formation [26,27,28], and further research is needed to elucidate the interplay between hemoglobin levels, renal oxygenation, and stone pathogenesis.

A higher preoperative stone burden—defined as the sum of stone diameters in three dimensions—was independently associated with recurrence in logistic regression and machine learning analyses. This consistent finding strongly supports the association between the stone burden and the risk of stone recurrence [29]. The strong association between a larger stone size and the recurrence risk may reflect prolonged exposure to a lithogenic environment, often driven by persistent metabolic abnormalities such as hypercalciuria, hyperoxaluria, and obesity [8,19,30]. These conditions promote sustained crystal deposition and may persist even after stone removal. In addition, anatomical factors, such as narrow infundibula, steep calyceal angles, or ureteropelvic junction obstruction, can impair stone clearance [31]. The stone size may serve as a surrogate marker for both metabolic and anatomical risk factors involved in stone recurrence. Although our model included basic CT features (the calyceal number and hydronephrosis), these did not emerge among the top predictors, likely due to our modest sample size and lack of detailed 3D anatomical assessment. Future studies employing 3D CT segmentation to assess the renal pelvis, calyces, and ureter morphology may enable further precision in risk stratification.

In addition to anatomical imaging features, we also examined intraoperative parameters that are unique to ECIRS. Our dataset included ECIRS-specific intraoperative parameters, such as the surgery time, presence of ureteral injury, and laser usage, across eight variables (see Supplementary Table S1). However, none of these emerged as strong predictors of recurrence in SHAP analysis. This suggests that the recurrence risk is more influenced by metabolic abnormalities and individual patient profiles than by intraoperative factors alone. Accordingly, the developed model may also apply to other stone surgeries, including PCNL or retrograde intrarenal surgery, provided baseline and follow-up data are available. These findings underscore the importance of an accurate recurrence prediction in high-risk surgical populations. Patients undergoing surgery often harbor multiple risk factors for stone growth and are thus more prone to recurrence than those managed conservatively. Yet, no established prediction models or preventive strategies exist, reinforcing the need for tools that can guide personalized postoperative management.

Despite its strengths, our study has limitations. It was conducted at a single tertiary center with a relatively small cohort, which limited the external validation and may affect the generalizability. The applicability is restricted to calcium stone formers; external validation in non-calcium stone cohorts is required. While we acquired complete data, the real-world use of 24 h urine testing may be constrained by patient compliance, cost, and variability. A single urine sample may not capture day-to-day fluctuations due to diet or hydration. Further, although machine learning improves prediction, it may be viewed as a “black box.” We mitigated this by using SHAP values for transparency, but simplified clinical calculators may be needed for broader adoption.

In conclusion, our study supports a paradigm shift in stone management from general recurrence estimation to personalized postoperative risk stratification. By leveraging rich datasets and modern AI tools, we identified previously underappreciated markers of recurrence risk, such as urinary oxalate excretion and postoperative hemoglobin trends, even within normal ranges. These findings support the integration of metabolic, anatomical, and physiological insights into postoperative care strategies and may inform future guidelines. Larger, multicenter studies and external validation are warranted to confirm and expand upon these results.

## 5. Conclusions

This study demonstrated that machine learning models can accurately predict postoperative kidney stone recurrence after ECIRS. Incorporating 24 h urine data improved both the risk prediction and reclassification performance. Notably, even patients with oxalate excretion levels considered within the normal range were at increased risk of recurrence, highlighting the need to re-evaluate current thresholds in postoperative care.

## Figures and Tables

**Table 1:** Comparison of baseline characteristics and 24 h urine parameters between patients with and without stone recurrence.

Characteristics	With Recurrence (*n* = 29)	Non-Recurrence (*n* = 43)	*p* Value *
Sex, men, No (%)	12 (41.4)	12 (27.9)	0.35
Age, Mean ± SD (years)	61.8 ± 10.1	61.7 ± 12.8	0.94
BMI, Mean ± SD (kg/m^2^)	25.0 ± 5.1	25.5 ± 5.4	0.70
**Medical history**			
Kidney stone history (*n*)	1.5 ± 1.6	0.9 ± 1.5	0.11
Age at first stone, Mean ± SD (years)	46.6 ± 12.9	51.4 ± 13.2	<0.05
ESWL history, Mean ± SD (*n*)	0.3 ± 0.5	0.2 ± 0.4	0.47
TUL history, Mean ± SD (*n*)	0.3 ± 0.5	0.1 ± 0.3	<0.05
PCNL history, Mean ± SD (*n*)	0.2 ± 0.4	0.0 ± 0.2	<0.05
**Stone-Related Factors**			
Side, right, No (%)	17 (58.6)	27 (62.8)	0.91
Bilateral, No (%)	10 (34.5)	3 (7.0)	<0.05
Total stones, Mean ± SD (*n*)	1.9 ± 1.1	1.4 ± 0.8	<0.05
CT value	1414.2 ± 348	1347.5 ± 235	0.37
Stone composition, CaOX, No (%)	20 (68.9)	42 (97.7)	<0.05
**24 h urine collection test**			
Volume, Mean ± SD (ml/day)	1998.3 ± 777	1733.7 ± 999	0.21
pH	6.3 ± 0.9	6.1 ± 0.8	0.13
Creatinine, Mean ± SD (g/day)	1.1 ± 0.4	1.4 ± 1.2	0.11
Uric Acid, Mean ± SD (g/day)	0.52 ± 0.21	0.58 ± 0.44	0.47
Calcium, Mean ± SD (g/day)	0.18 ± 0.09	0.19 ± 0.14	0.60
Magnesium, Mean ± SD (mg/day)	72.8 ± 27.6	88.4 ± 58.2	0.13
Oxalate, Mean ± SD (mg/day)	35.9 ± 19.7	26.9 ± 17.0	<0.05
Phosphate, Mean ± SD (g/day)	0.70 ± 0.29	0.87 ± 0.82	0.23

* *p* values were calculated using the Chi-square test, independent samples *t*-test, or Mann–Whitney U test, as appropriate.

**Table 2:** Univariable and multivariable logistic regression analyses for predictors of postoperative stone recurrence.

	Univariable		Multivariable	
Characteristics	OR (95%CI)	*p* Value	OR (95%CI)	*p* Value
Age	1.00 (0.96–1.04)	0.962		
Sex, men	1.82 (0.67–4.93)	0.236		
Bilateral	7.02 (1.73–28.49)	0.006	12.0 (3.5–41.2)	0.001
stone burden (per 10 mm)	1.22 (1.00–1.63)	0.028	1.22 (1.10–1.79)	0.013
Oxalate(24 h urine) (per 10 mg/day)	1.34 (0.90–1.79)	0.057	1.48 (1.10–2.16)	0.021

**Table 3:** Comparison of predictive performance across machine learning and deep learning models for postoperative stone recurrence.

Model *		Accuracy	Sensitivity	Specificity	F1 Score	ROC-AUC
ML	Random forest	70.7 (±5.9)	55.5 (±15.0)	70.7 (±19.6)	0.59 (±0.100)	0.74 (±0.080)
	LightGBM	66.7 (±2.6)	44.6 (±4.6)	81.4 (±5.7)	0.52 (±0.040)	0.66 (±0.071)
	XGBoost	70.9 (±5.0)	53.1 (±13.8)	84.6 (±9.8)	0.59 (±0.069)	0.75 (±0.065)
DL	AutoINT	73.4 (±9.7)	61.3 (±10.7)	81.4 (±11.4)	0.65 (±0.130)	0.76 (±0.097)
	TabNET	80.5 (±6.2)	80.0 (±11.5)	81.4 (±13.3)	0.77 (±0.028)	0.89 (±0.021)

* Values represent the mean ± standard deviation calculated from 5-fold cross-validation.

**Table 4:** Comparison of predictive performance across model-based combinations of clinical tests.

	Accuracy	Sensitivity	Specificity	F1 Score	ROC-AUC
Model 1	80.5 (±6.2)	80.0 (±11.5)	81.4 (±13.3)	0.77 (±0.028)	0.89 (±0.021)
Model 2	79.2 (±10.5)	76.0 (±23.3)	81.7 (±11.8)	0.73 (±0.151)	0.84 (±0.100)
Model 3	73.5 (±17.6)	86.0 (±17.7)	66.4 (±13.9)	0.68 (±0.120)	0.79 (±0.224)
Model 4	73.1 (±8.9)	62.1 (±16.2)	79.7 (±16.2)	0.66 (±0.115)	0.75 (±0.137)
Model 5	72.3 (±5.9)	53.0 (±13.3)	86.1 (±9.0)	0.60 (±0.090)	0.69 (±0.071)

## Data Availability

The data presented in this study are available on request from the corresponding author. However, they are not publicly available due to privacy concerns and restrictions based on informed consent. Data sharing is not permitted without additional approval from the institutional review board and ethical committee.

## Supplementary Materials

The following supporting information can be downloaded at: https://www.mdpi.com/article/10.3390/jcm14124037/s1, Table S1: List of clinical and laboratory variables used for prediction modeling.

## Abbreviations

The following abbreviations are used in this manuscript:

AI	Artificial intelligence
AUC-ROC	Area under curve–receiver operating characteristics
AutoInt	Automatic feature interaction learning via self-attentive neural networks
BMI	Body Mass Index
CI	Confidence interval
CT	Computed tomography
DL	Deep learning
ECIRS	Endoscopic combined intrarenal surgery
Hb	Hemoglobin
HU	Hounsfield units
IDI	Integrated Discrimination Improvement
LightGBM	Light Gradient-Boosting Machine
ML	Machine learning
NRI	Net Reclassification Improvement
PCNL	Percutaneous nephrolithotomy
SFR	Stone-free rate
SHAP	SHapley Additive exPlanations
TabNet	Tabular data learning network
URS	Ureteroscopy
XGBoost	eXtreme Gradient Boosting
