# Supplementary material for: AI-Driven Prediction of Renal Stone Recurrence Following ECIRS: A Machine Learning Approach to Postoperative Risk Stratification Incorporating 24-Hour Urine Data
Source: J Clin Med. 2025 Jun 7;14(12):4037. doi: 10.3390/jcm14124037 (PMC12193965; doi:10.3390/jcm14124037)
Supplement: Supplementary file 1 [file jcm-14-04037-s001.zip › jcm-3661290-supplementary.pdf]

Supplementary Table S1. List of clinical and laboratory variables used for prediction modeling

	Column Name		Column Name
**Initial visit**	**Demographics**	**Postoperative day 1**	**Blood test**
	age (years)		WBC count (/μL)
	sex		Hemoglobin (g/dL)
	side		Hematocrit (%)
	height (cm)		Blood Urea Nitrogen(mg/dL)
	weight (kg)		Creatinine(mg/dL)
	BMI (kg/m²)		C-Reactive Protein(mg/dL)
	Performance Status		Sodium, Na (mEq/L)
	diabetes mellitus		Potassium, K (mEq/L)
	hypertension		Chloride, Cl (mEq/L)
	hyperlipidemia		Calcium, Ca (mg/dL)
	heart disease		Uric Acid, UA (mg/dL)
	cerebrovascular disease		Albumin (g/dL)
	history of urolithiasis		**Urine test**
	osteoporosis		gravity
	calcium channel blocker use		pH
	angiotensin-converting enzyme inhibitor (blood pressure medication) use		protein
	angiotensin II receptor blocker (blood pressure medication) use		glucose
	diuretic use		RBC (/HPF)
	oral antidiabetic drug use		WBC (/HPF)
	insulin use		bacteriuria
	steroid use		urine culture
	uric acid lowering drug use		**Complication**
	lipid-lowering drug use		post pain
	antiplatelet drug use		post fever (≥38˚C)
	anticoagulant drug use		post septic
	vitamin D supplement use	**Postoperative 1 month**	**Urine test**

	bisphosphonate use		gravity
	alpha-1 blocker use		pH
	anticholinergic agent use		protein
	beta-3 agonist use		glucose
	age at first urolithiasis episode		RBC (/HPF)
	number of urolithiasis episodes		WBC (/HPF)
	history of Shock Wave Lithotripsy (SWL)		bacteriuria
	Ureteroscopy (URS) history		urine culture
	Percutaneous Nephrolithotomy (PCNL) history		**Complication**
	history of open stone surgery		complications (grade)
	history of abdominal surgery		pain
	family history of urolithiasis		fever (≥38˚C)
	smoking habit		septic
	alcohol consumption		hematuria
	**Chief complaint (symptomatic)**		stone passage
	pyelonephritis		urinary tract infection
	Screening Abnormalities		**Add treatment**
	hematuria		analgesics
	pain		antibiotics
	antibiotics		medical expulsive therapy
	double-J stent placement		double-J stent placement
	percutaneous nephrostomy		percutaneous nephrostomy
	**Blood test**		SWL
	WBC count (/µL)		URS
	Hemoglobin (g/dL)		PCNL
	Hematocrit (%)		stone composition (CaOx/CaP)
	Blood Urea Nitrogen(mg/dL)	**Postoperative 3 Months**	**Blood test**
	Creatinine(mg/dL)		WBC count (/µL)
	C-Reactive Protein(mg/dL)		Hemoglobin (g/dL)
	Sodium, Na (mEq/L)		Hematocrit (%)
	Potassium, K (mEq/L)		Blood Urea Nitrogen(mg/dL)
	Chloride, Cl (mEq/L)		Creatinine(mg/dL)

	Calcium, Ca(mg/dL)		C-Reactive Protein(mg/dL)
	Uric Acid, UA (mg/dL)		Sodium, Na (mEq/L)
	Albumin (g/dL)		Potassium, K (mEq/L)
	**Urine test**		Chloride, Cl (mEq/L)
	gravity		Calcium, Ca (mg/dL)
	pH		Uric Acid, UA (mg/dL)
	protein		Albumin (g/dL)
	glucose		**Urine test**
	RBC (/HPF)		gravity
	WBC (/HPF)		pH
	bacteriuria		protein
	urine culture		glucose
	**Clinical manifestations**		RBC (/HPF)
	pyelonephritis		WBC (/HPF)
	pain		bacteriuria
	Screening Abnormalities		urine culture
	hematuria		**Complication**
	**Treatment for stones**		complications (grade)
	medical expulsive therapy		pain
	analgesics		fever (≥38℃)
	antibiotics		septic
	double-J stent placement		hematuria
	percutaneous nephrostomy		stone passage
	**CT imaging**		urinary tract infection
	total number of stones		**Add treatment**
	number of stones on the right side		analgesics
	number of stones on the left side		antibiotics
	location of individual stones		medical expulsive therapy
	lower caliceal stone		double-J stent placement
	stone density（HU）		percutaneous nephrostomy
	stone dimensions (length × width × height, mm)		SWL
	maximum stone diameter (mm)		URS
	stone burden(mm)		PCNL

	volume of individual stones	**Postoperative 6 Months**	**24-hour urine collection**
	stone total volume		urine volume (ml/day)
	number of stone branches		Sodium, Na (mEq/day)
	Hydronephrosis (grade)		Potassium, K (mEq/day)
	staghorn stone (Complete / Partial / None)		Chloride, Cl (mEq/day)
	single kidney		Magnesium, Mg (mg/day)
**Surgery**	**Surgical details**		Calcium, Ca (mg/day)
	surgery time		Phosphorus, P (g/day)
	endoscopic residual stones		Uric Acid, UA (mg/day)
	ureteral damage		Creatinine (g/day)
	postoperative percutaneous nephrostomy insertion		Oxalate (mg/day)
	postoperative double-J stenting		supersaturation of calcium oxalate
	lihocrast use		supersaturation of calcium phosphate
	laser use		
	ultrasonic use		
